# Emerging Antifungal Targets and Strategies

**DOI:** 10.3390/ijms23052756

**Published:** 2022-03-02

**Authors:** Marija Ivanov, Ana Ćirić, Dejan Stojković

**Affiliations:** Department of Plant Physiology, Institute for Biological Research “Siniša Stanković”—National Institute of Republic of Serbia, University of Belgrade, Bulevar despota Stefana 142, 11000 Belgrade, Serbia; rancic@ibiss.bg.ac.rs (A.Ć.); dejanbio@ibiss.bg.ac.rs (D.S.)

**Keywords:** antifungal targets, drug discovery, antibiofilm, mitochondrial activity, fungal resistance, *Candida*, *Aspregillus*, mechanism of action

## Abstract

Despite abundant research in the field of antifungal drug discovery, fungal infections remain a significant healthcare burden. There is an emerging need for the development of novel antifungals since those currently available are limited and do not completely provide safe and secure protection. Since the current knowledge regarding the physiology of fungal cells and the infection mechanisms is greater than ever, we have the opportunity to use this for the development of novel generations of antifungals. In this review, we selected and summarized recent studies describing agents employing different antifungal mechanisms. These mechanisms include interference with fungal resistance, including impact on the efflux pumps and heat shock protein 90. Additionally, interference with virulence factors, such as biofilms and hyphae; the impact on fungal enzymes, metabolism, mitochondria, and cell wall; and antifungal vaccines are explored. The agents investigated belong to different classes of natural or synthetic molecules with significant attention given also to plant extracts. The efficacy of these antifungals has been studied mainly in vitro with some in vivo, and clinical studies are needed. Nevertheless, there is a large quantity of products employing novel antifungal mechanisms that can be further explored for the development of new generation of antifungals.

## 1. Fungi and Fungal Infections

Fungi are eukaryotic unicellular or multicellular organisms. They have a specific cell wall structure and cytoplasmic membrane made of sterols, primarily ergosterol. Fungi causing disease in humans morphologically can be yeasts, molds, and biphasic (dimorphic) fungi [1]. It is estimated that fungal infections, primarily the ones associated with *Candida*, *Cryptococcus* and *Aspergillus* species, kill more than one million people each year. These underestimated diseases are challenging to eradicate, and the linked mortality is very high even despite the existence of antifungal treatments [2].

Depending on the type of human mycosis, they can be exogenous (e.g., dermatomycoses), endogenous (e.g., candidiasis), and exo-endogenous (e.g., cryptococcosis).

According to the localization, mycosis can be:

(a) Superficial (superficial, cutaneous, and mucocutaneous). Infections are most common on the nail (onychomycosis), eye (keratomycosis), and ear (otomycosis).
(b) Deep/invasive—fungi are localized and multiply in deep tissues and organs (lungs, liver, spleen, bones, brain, heart, and blood). These infections have a complex clinical course, poor prognosis, and are difficult to diagnose. They are most often caused by fungi from the genera *Aspergillus* and *Candida* [1,3,4].

## 2. Current Antifungals in Use and Their Limitations

Antimycotics/antifungals are drugs used in the treatment of fungal infections; however, their number is limited and is significantly lower compared to drugs used to treat bacterial infections [5].

Therapy of fungal infections is difficult and time consuming. Fungi are eukaryotic microorganisms, and many cellular and molecular processes take place in a similar way as in human cells. Therefore, it is difficult to create drugs with high selective potential and low side effects on human cells [5].

Antimycotics are drugs for the treatment of fungal infections, and the most commonly used are [6]:

- Polyenes (amphotericin B, natamycin, and nystatin).
- Azoles—imidazoles (ketoconazole and miconazole) and triazoles (fluconazole, itraconazole, voriconazole, etc.).
- Echinocandines.
- Amines (terbinafine, etc.).

These drugs are used for local or systemic applications. 

### 2.1. The Most Common Antifungal Drugs for Topical Applications

**Figure 1:**
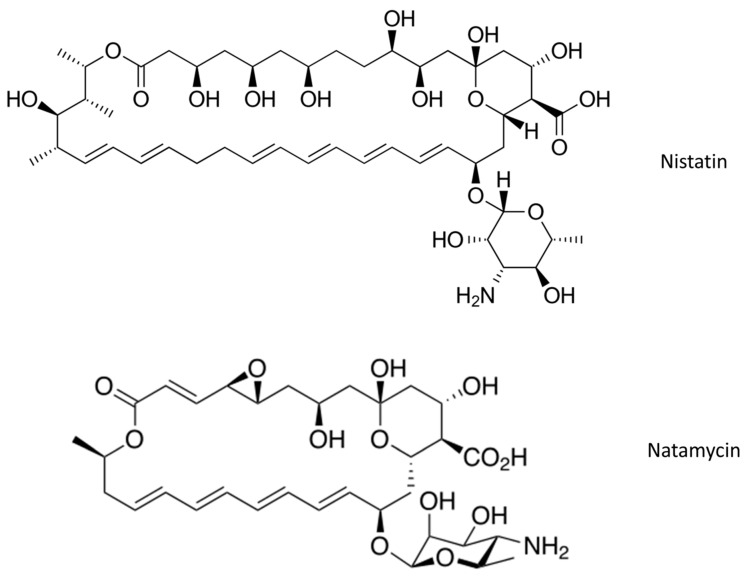
Chemical structures of nystatin and natamycin.

The most common antifungal drugs [7,8,9] for topical application from the class of polyene macrolides include nystatin and natamycin (Figure 1). 

Nystatin is a polyene macrolide derived from the mold *Streptomyces noursei*. It has a fungistatic and fungicidal effect on a number of fungi in vitro. Due to its characteristic structure, it binds to ergosterol molecules in the fungal wall membrane and causes cavities in the membrane (until then, the impermeable membrane becomes very permeable and loses its function), which destroys the integrity of the fungal wall and finally causes its death. Nystatin has a fungistatic and fungicidal effect on saprobic and parasitic yeasts especially against *Candida* spp. It is used in the treatment of local candidiasis in the corners of the lips; mucous membranes of the oral cavity; intestines; space between fingers, nails, anorectal area, and outer ear; and prophylaxis of candidiasis in newborns [10]. 

Side effects of nystatin include mild indigestion (diarrhea, nausea, vomiting, and abdominal pain) that may rarely occur during oral nystatin use. Irritation of the skin and mucous membranes may occur at the site of use and less frequently skin signs of hypersensitivity (maculopapular rash) [10].

Natamycin is a polyene antifungal; it accomplishes its antifungal effect by specific binding to ergosterol while not increasing permeability of the cell membrane [11]. Natamycin induces ergosterol-specific and reversible inhibition of membrane transport proteins, and by inhibiting membrane transport it eventually inhibits fungal growth [12]. Side effects include chest pain and dyspnea [13].

**Figure 2:**
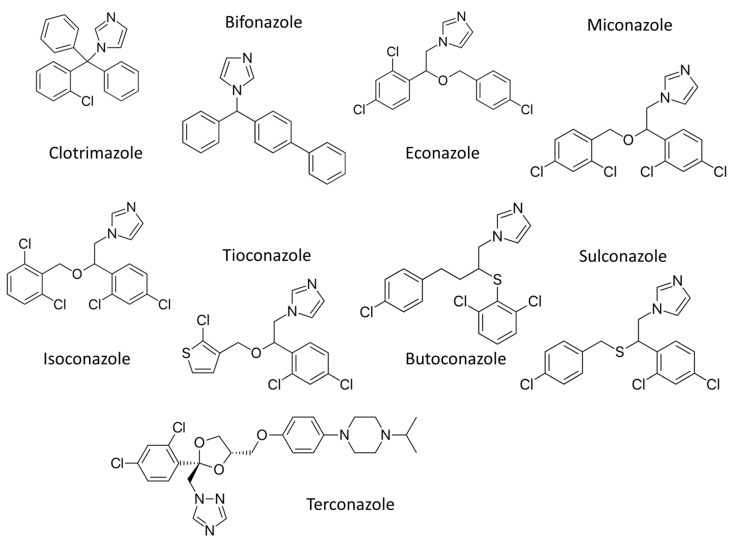
Chemical structures of antifungal azoles for topical applications.

The most common antifungal drugs for topical application from the azole class [7] include derivatives of imidazole (clotrimazole, bifonazole, econazole, miconazole, isoconazole, tioconazole, butoconazole, and sulconazole) and derivatives of 1,2,4 –triazole (terconazole) (Figure 2). The mechanism of activity for the drugs from azole class is the inhibition of the ergosterol synthesis by direct binding to sterol 14α-demethylase (CYP51). The enzyme CYP51 is present in the endoplasmic reticulum outer membrane and its role is catalyzation of the removal of the methyl group at carbon 14. 

Azole binding to the active site of CYP51 is non-competitive and induces a depletion of the final biosynthetic product—ergosterol—while it also induces accumulation of 14-methylated sterols that inhibit fungal growth by reducing the integrity of the cell membrane [14]. The fungal cells are able to induce resistance to azole treatment through the upregulation of drug transporters. Upregulations of *CDR1*/*CDR2* and *MDR1* are achieved through point mutations in *TAC1* and *MRR1* transcription factors. 

Likewise, fungal cells can reduce lanosterol 14-α-demethylase binding affinity for the drug via point mutations in *ERG11* and can also increase concentration of target enzyme by *ERG11* upregulation. Additionally, point mutations in *ERG3* can inactivate C5 sterol desaturase leading to alterations in the ergosterol synthetic pathway and inducing azole resistance [15]. We are witnessing the increase in azole resistant fungal strains worldwide. For example, in the Netherlands the incidence of *Aspergillusfumigatus* resistant strains increased from 0.79% for 1996–2001 to 7.04% for 2012–2016 [16].

Clotrimazole is an imidazole antifungal drug with a broad spectrum of action and is one of the most widely used antifungals. In smaller doses, it has fungistatic and in higher doses fungicidal action on *Candida albicans*, *Trichophyton rubrum*, *Trichophyton mentagrophytes*, *Epidermophyton floccosum*, *Microsporum canis,* and *Malassezia furfur* [17]. 

Side effects and limitations of use: Clotrimazole preparations should not come into contact with mucous membranes, nor should the patient expose the treated area to the sun. Irritation, burning, rash, itching, peeling, or skin edema may rarely occur at the site of use [17].

Miconazole is an imidazole derivative that has a fungicidal effect especially on dermatomycetes and *Candida*. It is intended for the treatment of skin infections caused by fungi of the genus *Candida*, treatment of *tinea pedis* (athletic foot), *tinea cruris* and *tinea corporis* caused by *Trichophyton rubrum* and *Trichophyton mentagrophytes*. It is also used to treat *Pityrosporum orbiculare* (*Malassezia furfur*) [18].

Side effects and limitations of use: It cannot be used in the form of tablets or capsules because it is not absorbed enough from the digestive tract. If it is given parenterally (by injection), it may cause many side effects, and thus it can only be given in emergency and life-threatening situations. When using miconazole in the form of a cream, sporadic cases of maceration, allergic dermatitis, irritation, and redness of the skin have been described [18].

Ketoconazole, an imidazole derivative, is a broad-spectrum antifungal agent. It acts on dermatomycetes (*Microsporum*, *Trichophyton*, and *Epidermophyton*), yeasts (*Candida*, *Cryptococcus*, *Torulopsis*, and *Pityrosporum*), dimorphic fungi (*Histoplasma capsulatum*, *Coccidioides*, and *Paracoccidioides*), and eumycetes. It comes in the form of creams, shampoos, or tablets. In the form of a cream, it is used to treat skin fungal infections and seborrheic dermatitis. In the form of shampoo, it is used to treat and prevent pityriasis versicolor, seborrheic dermatitis, and dandruff. In tablet form, it is used to treat superficial and deep mycoses of the skin, scalp, and nails caused by dermatomycetes or candidiasis (dermatomycosis, onychomycosis, chronic mucocutaneous candidiasis, pityriasis versicolor, etc.), oral and digestive candidiasis, and as a systemic antifungal [19].

Side effects and limitations of use: While the use of ketoconazole as a cream and shampoo is generally not dangerous, the use of ketoconazole in tablet form can be dangerous because it can cause liver dysfunction. Therefore, the use of ketoconazole in patients with impaired renal or hepatic function is prohibited, as well as the use of ketoconazole tablets in pregnant women. Topical application of ketoconazole may, although rarely, cause skin or scalp irritation or burning, and hair may become oily or dry. The active substance and auxiliary ingredients in the cream sometimes cause a local allergic reaction (contact dermatitis) [19]. 

**Figure 3:**
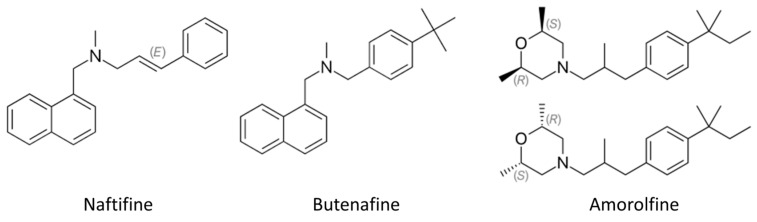
Chemical structure of antifungal amines used for topical applications. (E)—trans isomer, (S)—sinister enantiomer, (R)—rectus enantiomer.

Amines are mainly used for the topical treatment of fungal infections [8]. These drugs include naftifine, butenafine, and amorolfine (Figure 3). The allylamines inhibit synthesis of a fungal lipid, ergosterol, by targeting squalene epoxidase. Squalene epoxidase is an essential flavin adenine dinucleotide-dependent enzyme of ergosterol and cholesterol biosynthetic pathways with a role in catalyzing the stereospecific epoxidation of squalene to 2,3-(S)-oxidosqualene [20]. This class of antifungals is selective for the fungal enzyme with minimal impact on cholesterol synthesis in mammals. While naftifine possesses only topical activity, its analog terbinafine is active both topically and orally [21].

Side effects when applied topically may occur, such as burning, stinging, irritation, redness, dry skin, or itching at the application site.

Organic acids and their derivatives are used as well as antifungals for topical application and include sorbic acid and its salts, undecylenic acid, propionic acid, caprilic acid, triacetin, and salicylic acid [22]. 

### 2.2. The Most Common Antifungal Drugs for Systemic Applications

5-fluorocytosine is a pyrimidine antifungal drug and one of the oldest antifungals for the treatment of cryptococcosis, candidiasis, and chromoblastomycosis. This compounds antifungal action starts by entering into the fungal cells through permeases. 5-Fluorocytosine acts as a prodrug—upon entrance into the cell, it is converted to its metabolically active form 5-fluorouracil by cytosine deaminase. 5-Fluorouracil can be converted to 5-fluorouridine monophosphate or 5-fluorodeoxyuridine monophosphate and inhibit DNA replication, transcription, and protein synthesis. Unfortunately, this agent is not suitable as monotherapy for fungal infections since it provokes rapid development of resistance among fungal pathogens. It is used in combination with amphotericin B, the combination is the gold standard for induction therapy in cryptococcosis [23]. 

**Figure 4:**
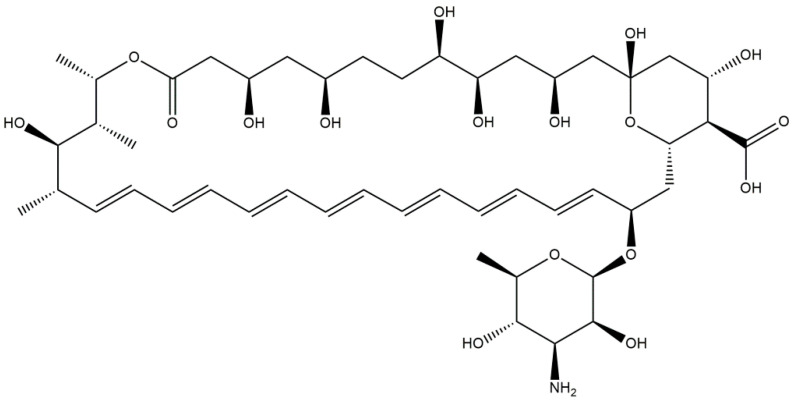
Chemical structure of Amphotericin B.

Amphotericin B (Figure 4) is, like nystatin, a polyene macrolide antifungal drug. It was obtained from a culture of the fungus *Streptomyces nodosus*. The mechanism of action is identical to the mechanism of action of nystatin—interaction with ergosterol in the fungal membrane by creating pores in the cell membrane [24]. Amphotericin B interacts with the membrane lipid bilayer through its hydrophobic domains, which induces formation of multimeric pores along with subsequent increase in the permeability of ions (K^+^, Ca^2+^, and Mg^2+^). Eventually, intracellular loss induces cell death [25]. On the other hand, it is also suggested that Amphotericin B is mainly in the form of large, extramembranous aggregates that extract ergosterol from lipid bilayers as its antifungal mechanism. It is proposed that extraction of the essential cell lipid underlies the antifungal action of this compound [26].

Side effects: This antifungal drug is poisonous to humans; however, when it comes to systemic mycoses, we follow the principle of choosing the lesser of two evils. Namely, it is important that the benefit to the patient outweighs the risk. It is used in the form of infusions, and the treatment lasts for several weeks [24]. The formulation initially developed in the 1950s was amphotericin B deoxycholate. However, due to recorded significant dose-limiting toxicity of this formulation, mainly nephrotoxicity and infusion-related reactions, less toxic formulations were developed [27]. Different lipid formulations of amphotericin B are available, including AmBisome^®^ (liposomal amphotericin B), Abelcet^®^ (amphotericin B lipid complex), and Amphocil/Amphotec^®^ (amphotericin B colloidal dispersion). Liposomal formulation has less toxicity compared to other lipid formulations, and can be safely and repeatedly applied at high concentrations for treating infections that are challenging to cure, such as fusariosis and zygomycosis [28]. 

**Figure 5:**
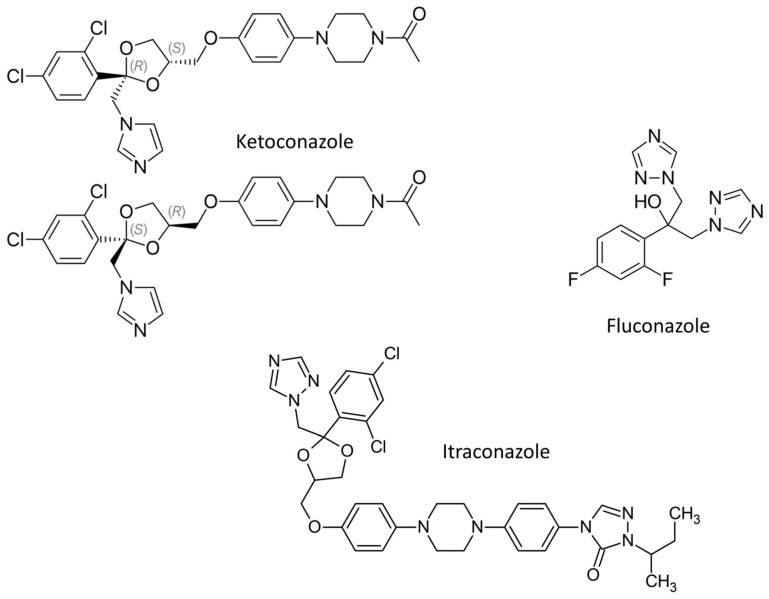
Chemical structure of azole derivatives used as systemic antifungal drugs. (S)—sinister enantiomer, (R)—rectus enantiomer.

Ketoconazole (Figure 5), an imidazole derivative, is effective in systemic mycoses except in aspergillosis (infection with the fungus *Aspergillus* sp.). Side effects: The use of ketoconazole in tablet form can cause several side effects—digestive disorders, nausea, dizziness, headache, photophobia, paresthesia, thrombocytopenia, and exanthema are rare. Alopecia, urticaria, or any allergic rash is very rare, and fontanelle tension occurs in infants. An increase in liver enzyme activity has also been observed and, exceptionally, hepatitis. In some cases, when higher doses (more than 400 mg) were prescribed, gynecomastia and oligospermia occurred. Sometimes testosterone levels dropped temporarily. The disadvantage of systemic ketoconazole administration is marked hepatotoxicity (toxicity to the liver). It is also important to emphasize that ketoconazole is a proven teratogen and that pregnant women should never use ketoconazole [19].

Fluconazole is by chemical structure bistriazole, a derivative of 1,2,4-triazole (Figure 5). It is important in the treatment of meningitis caused by fungi and also in patients with impaired immune systems. It can also be used in the treatment of fungal skin diseases and is especially effective in vaginal candidiasis. Its toxicity is far less than that of miconazole and ketoconazole [29].

Itraconazole is a 1,2,4-triazole derivative (Figure 5). The greatest value of itraconazole is that it is concentrated in tissues, especially those that are prone to fungal infections. Therefore, it is extremely effective not only in systemic mycoses (systemic aspergillosis and candidiasis, cryptococcosis including cryptococcal meningitis, histoplasmosis, sporotrichosis, paracoccidioidomycosis, and blastomycosis) but also in skin diseases. Unfortunately, it is not active in brain tissue [30,31].

Posaconazole is a second-generation triazole with an extended-spectrum. Its long side chain facilitates hydrophobic binding to the target enzyme CYP51 and is the reason posaconazole is active against many fluconazole and voriconazole resistant strains. Its active against *Candida*, *Aspergillus,* and *Mucorales* [32]. The drug absorption is enhanced when posaconazole is given with food, nutritional supplements, and carbonated beverages, while the oral administration in divided doses raises its bioavailability. The most frequently reported side effects are headache, fatigue, nausea, vomiting, and elevated hepatic enzymes [33]. 

Voriconazole is a second-generation triazole antifungal drug active against *Candida*, *Aspergillus*, *Scedosporium*, and *Fusarium* spp. It is the first-line treatment for invasive aspergillosis and is used also in therapies for *Candida* spp. The broad use of this compound has been narrowed due to the recorded high frequency of adverse effects including neurotoxicity, visual toxicity, and hepatotoxicity [34]. Additionally, the most concerning side effect of voriconazole use is the associated increased risk of cutaneous malignancies, primarily squamous cell carcinoma [35]. 

**Figure 6:**
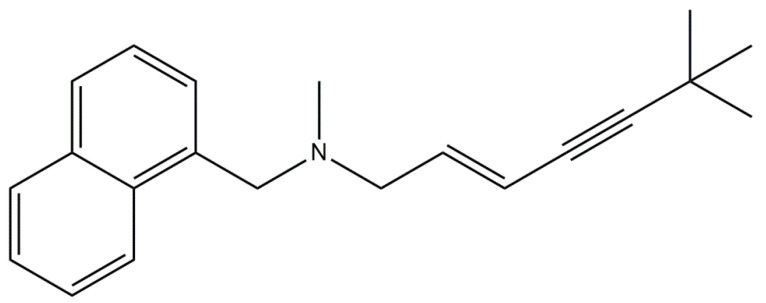
Chemical structure of Terbinafine.

Terbinafine (Figure 6), an amine antifungal drug, is a unique antimycotic. At low concentrations, terbinafine has a fungicidal effect on dermatophytes, molds, and some dimorphic fungi. It has a fungicidal or fungistatic effect on yeasts, depending on the species. Terbinafine specifically interferes with fungal sterol biosynthesis in its early stages. This leads to ergosterol deficiency and intracellular accumulation of squalene, resulting in fungal cell death. Terbinafine acts by inhibiting squalene epoxidase in the fungal cell membrane. The enzyme squalene epoxidase is not bound to the cytochrome P450 system. Therapy lasts shorter than with other antifungal drugs. When taken orally, the drug is concentrated in the skin, hair, and nails in fungicidal concentrations. In this way, these organs become saturated with terbinafine and are very effective in relieving fungal infections. Therapy lasts shorter than with other antifungal drugs—when terbinafine is administered in the form of a cream, the withdrawal of clinical symptoms usually occurs within a few days, and therapy lasts up to two weeks [36].

Side effects and limitations: Terbinafine, administered in tablet form, interferes with renal and hepatic function and should therefore be used to a very limited extent in patients with impaired renal and hepatic function. The most common side effects of terbinafine tablets are digestive symptoms (bloating, loss of appetite, nausea, mild abdominal pain, and diarrhea) or harmless forms of skin reactions (redness and urticaria), musculoskeletal reactions (arthralgia and myalgia), and neutropenia [37,38].

Echinocandins are a class of antifungals with a cyclic non-ribosomal hexapeptides and a lipophilic side chain. These compounds are produced by filamentous fungi and have been used for the development of semisynthetic derivatives, such as caspofungin, micafungin, and anidulafungin, a first-line antimycotics for the treatment of invasive mycosis. Their mechanism of activity is based on the inhibition of 1,3-β-D-glucan synthase, which subsequently interferes the synthesis of β-(1,3) d-glucan, an essential cell wall polysaccharide [39]. There has been an increase in the incidence of *Candida* species resistant to echinocandins. The resistance is associated with mutations in *FKS1* and *FKS2* genes. The exact mechanism of resistance is linked with the mutation of two regions of the FKS1p subunit of β-(1,3) d-glucan synthase, which subsequently leads to the substitution of serine 645 for proline, phenylalanine, and tyrosine and changes the echinocandins target site [40].

## 3. Recent Update on Antifungal Targets and Strategies

Over the past few years, the significant progress has been achieved in the field of antifungal drug discovery. However, since limited percentage of compounds that are currently undergoing clinical studies will be finally approved for further human use, it is necessary that additional antifungal compounds are detected and explored at this moment and in the future [41].

In the last few years, our knowledge regarding the structural and functional components of fungal cells has drastically increased. Now, we are more than ever familiar with the diverse spectrum of virulence factors that fungal cells utilize in order to establish infections. Among the virulence factors, biofilms have been extensively studied, and we became familiar with their architecture and mechanisms underlying their establishment. All this knowledge can be exploited in order to develop antifungals that would be oriented towards some novel antifungal targets. 

The review by McCarthy et al. [42] indicated that different structures of fungal cells could be targeted in order to develop novel antifungals including cell wall and cell membrane; but also metabolic pathways (glyoxylate cycle, pyrimidine biosynthesis, cytochrome P450 enzymes, iron metabolism, heme biosynthesis, and acetate metabolism), signal transduction pathways (MAP kinase, PDK1, and calcium signaling), and gene expression [42]. Some unconventional pathways were also recently proposed as possible targets for the antifungal development: enolase, a part of the enolase-plasminogen complex, along with enzymes involved in the mannitol biosynthesis and purine nucleotide biosynthesis [43]. 

The antifungal targets presented in this review include fungal resistance factors, such as efflux pumps and heat shock protein 90, and fungal virulence factors, such as biofilms and hyphae. Additionally, fungal enzymes, metabolism, mitochondria, and cell walls as antifungal targets were explored, as well as antifungal vaccines. 

### 3.1. Interference with Fungal Antimicrobial Resistance

Currently, we are witnessing an increase in antifungal resistance rates among the pathogenic fungi. For example, the study in Bari, Italy has recorded increasing rate of fluconazole resistant *C. albicans* strains isolated in their hospital (from 2.3% in 2015 to 14.2% in 2018) [44]. Likewise, multidrug-resistant yeast *Candida auris* some time ago emerged worldwide and became a significant threat due to its ability to rapidly develop resistance [45]. One study indicated that 79.6% of *C. auris* isolates are resistant to fluconazole, while 34.8% and 23.3% are resistant to amphotericin B and voriconazole, respectively [46]. 

Fungi belonging to *Candida*, *Aspergillus*, *Cryptococcus*, and *Pneumocystis* are generally recognized as the fungal pathogens with significant rates of antifungal resistance [47]. Antimicrobial resistance in fungal kingdom can be induced by different factors. Mechanisms of antifungal resistance include decrease of effective drug concentration, drug target alterations, and metabolic bypasses [48]. These mechanisms could be tangled by some novel agents and could be used for the development of novel therapeutic strategies based on the enhancement of the antifungal effect of current therapeutics. Recent reviews have suggested targeting fungal efflux pumps in order to combat drug resistance [49] as well as ergosterol biosynthesis pathway [50]. 

In the following section, we propose cell efflux, ergosterol biosynthesis, and Heat shock protein 90 (Hsp90) as potential targets for the development of antifungals that could reduce resistance mechanisms of pathogenic fungi (Table 1).

#### 3.1.1. Cell Efflux as Antifungal Target

One of the antifungal resistance mechanisms in *Candida* strains resistant to azole antifungals is upregulation of the ATP-binding cassette transporter genes *CDR1* and *CDR2* (Candida drug resistance 1 and 2) [98]. Azole resistance can be exceeded by inhibiting the pumps and subsequently again sensitizing resistant fungi to azole drugs. Novel agents that would target fungal efflux pumps could provide the basis for azole-enhancing combination therapy [49].

When seven flavonoids were studied for *CDR1* downregulation the most significant impact was accomplished by apigenin and apigetrin [56]. Likewise, astragalin was also able to downregulate *CDR1* [61]. Another flavonoid, kaempferol, could downregulate *CDR1* and *CDR2* genes in fluconazole-resistant *C. albicans* [99]. Berberine hydrochloride, traditional Chinese medicine with antimicrobial effects, reduces intracellular efflux of fluconazole by downregulating *CDR1* [100], while polyunsaturated fatty acids could reduce efflux from *C. krusei* cells [101]. Benzoxazole derivatives could interfere with both cell efflux and ergosterol synthesis in *C. albicans* [62].

#### 3.1.2. Ergosterol Biosynthesis and *ERG11* Expression

In yeast, the most abundant sterol is ergosterol. Sterols are essential for fungal cell maintenance since they coordinate membrane heterogeneity, prevent water penetration, and preserve the integrity, rigidity, and fluidity of the plasma membrane. The range of drugs targeting ergosterol biosynthesis present on the pharmaceutical market include azoles targeting lanosterol 14-α demethylase; polyenes drugs that disrupt the ergosterols distributed on the membrane; and allylamines that are squalene synthase (Erg1p) inhibitors [102]. 

Due to impact on the fungal cell viability and role as antifungal target, the ergosterol biosynthesis pathway is also involved in the resistance to antifungals. The overexpression of *ERG11* transcripts leads to the reduced azole susceptibility and can be the consequence of either by gain-of-function mutations in the transcriptional regulator, Upc2, or increased chromosome 5 copy number (on which *ERG11* resides). Mutations in *ERG11* are frequent among azole-resistant clinical fungal strains [103].

Apigenin, rutin, and palmitic acid downregulated the expression of *ERG11* [56,104]. Other studies targeted ergosterol biosynthesis by applying tormentic acid [105], benzodioxane derivatives [106], honokiol and magnolol [107], and cold atmospheric plasma [108].

#### 3.1.3. Heat Shock Protein 90 (Hsp90)

Hsp90 is molecular chaperone involved in the regulation of cellular signaling in eukaryotes. It is also included in drug resistance of both fungal biofilms and cells in the planktonic growth conditions [109], though targeting Hsp90 is observed as a potentially efficient antifungal strategy [110]. When four commercial Hsp90 inhibitors AUY922, ganetespib, PU-H71, and CH5138303 were evaluated for antifungal properties, the most promising activity was observed for ganetespib [111]. Although this compound did not show antifungal properties in the microdilution assay (MIC > 64 µg/mL), it has shown synergism when combined with fluconazole, which was also confirmed in vivo. This molecule also reduces cell efflux and downregulates expression of azole resistance linked genes, *CDR1*, *CDR2*, *ERG11*, and *MDR1*, when combined with fluconazole [111]. 

Sesquiterpene quinone, puupehenone, enhances the effect of caspofungin by disrupting Hsp90 activity and cell wall integrity in fungal pathogens [112]. Likewise, the synergistic effects of vorinostat with azoles against *Aspergillus* species is based on the Hsp90 suppression [113].

### 3.2. Tackling Different Virulence Aspects—Targeting Biofilm and Hyphae

Biofilms are communities established by fungal cells embedded in the extracellular matrix. By forming these structures fungal pathogens are able to escape being killed by immune cells, such as neutrophils and monocytes, making the eradication of biofilms a significant challenge [114]. A range of mechanisms is employed by fungi in order to increase biofilm resistance—high density of cells forming the structure, growth and nutrient limitation, the existence of persister cells, the expression of antifungal resistance gene and the increase of sterols content on the fungal cell membrane [115]. Biofilm resistance is also enhanced by the extracellular matrix due to its complex structure composed of proteins, carbohydrates, lipids, and extracellular DNA [116].

Many pathogenic fungi could alter their morphology—a trait that is usually tightly linked with their virulence. Morphological changes are a very habitual and operative strategy for numerous pathogens to survive in the mammalian organisms. The major fungal pathogens can grow in multiple morphologies, including yeast, pseudohyphae, and hyphae. The cAMP-PKA (cyclic adenosine monophosphate-protein kinase A) pathway and molecular chaperone Hsp90 pathways are used to control these morphological changes in many of the human pathogenic fungi [117]. 

For the leading human fungal pathogen *C. albicans*, the defining characteristic is its ability to switch between yeast, pseudohyphae, and hyphae during infection and disease. Hyphal form is serving as the scaffold for the biofilms and is linked with the microorganisms ability to induce infections [118]. Different products, including the ones from natural sources, have been studied so far due to their antibiofilm activities [119,120]; however, the search for efficient antibiofilm agents continues until today. 

Due to biofilm resistance, there is an urgent need for the development of antifungals that might cope with the ability of fungal pathogens to establish biofilms with recent studies highlighting potential biofilm inhibiting agents among range of different products (Table 1). Since the dimorphic characteristics could be a key of pathogenicity and virulence of many pathogenic fungi, the development of new, more potent antifungals targeting morphological transition is needed (Table 1).

Rosmarinic acid, a plant polyphenolic compound, could prevent biofilm formations through moderate reduction in exopolysaccharide (EPS) content in the biofilm matrix [89]. Likewise, cannabidiol, a non-psychoactive phytocannabinoid produced by *Cannabis sativa*, and usnic acid, a lichen secondary metabolite, reduce biofilm thickness and EPS production in *C. albicans* [67,94]. 

Extract from *Artemisia absinthium* displayed antibiofilm activities, as indicated by crystal violet assay, with reduction in EPS production possibly contributing to its antibiofilm effect. *A. absinthium* mechanism of antimicrobial activity is the disturbance of membrane integrity, as indicated by crystal violet uptake and nucleotide leakage assays [59]. Likewise, extract obtained from *Ononis spinosa* displayed antibiofilm potential towards three *Candida* strains, while its contributing antifungal mechanism was interference with membrane integrity [86].

The antimicrobial peptide VLL-28, isolated from an archaeal transcription factor, was able to significantly reduce fungal ability to form biofilms while its antifungal activity was based on damaging of the cell wall [55]. Photodynamic therapy could efficiently interfere with biofilms formed by *C. auris*, especially in combination with photosensitizing compounds [65]. Nanocomplex of the phytochemical curcumin and sophorolipid interferes with biofilm and hyphae of *C. albicans* by diverse mechanisms, including downregulation of virulence and resistance linked genes, such as hyphal regulatory genes *SAP4*, *HWP1,* and *HYR1,* as well as *RAS1* and *ERG11* [71]. 

Catechol, when applied in concentrations of 2–256 μg/mL, could dose-dependently inhibit biofilm and hyphal formation of *C. albicans*, it also inhibited virulence factors secreted hydrolases and reduced EPS levels in biofilm matrix. The qPCR data indicated that catechol application induces significant down-regulation of *RAS1*, *HWP1,* and *ALS3*, genes linked with the fungal pathogenesis [69].

The biological activity of selected flavonoids: flavone aglycones (luteolin and apigenin), a flavone glycosylated derivative (apigetrin), flavonol (quercetin), and its glycosylated derivatives (quercitrin, isoquercitrin, and rutin) in regulating the morphological switch between yeast and hyphal growth as well as their antibiofilm potential was studied [56]. The tested flavonoids possessed moderate activity in the terms of reducing fungal hyphal growth, with the most pronounced effect was observed for apigenin and apigetrin. The most pronounced antibiofilm activity at minimal inhibitory concentrations (MIC) was observed for isoquercitrin (76% inhibition) while both apigetrin and isoquercitrin applied in MIC/2 could prevent biofilm formation for more than 60% [56]. 

Further studies reports concerning anti-hyphal potential indicated that quercetin is more effective in concentration up to 200 µg/mL [121] and 64 µg/mL [122], while concentration of 75 µg/mL has induced only slight inhibition [56]. In another study, a concentration of 0.075 mg/mL of astragalin showed marginally reduced number of hyphal cells following 4 h treatment [61]. Morin was able to interfere with *C. albicans* hyphal growth, biofilm formation, phospholipase and exopolysaccharide production [82]. Likewise, dioscin has also displayed wide pallet of antifungal mechanisms including impairment of biofilm formation and development, morphological transition, adhesion, and extracellular secreted phospholipase [72]. 

Anti-hyphal potential of camphor (0.125 mg/mL) and eucalyptol (23 mg/mL) against clinical isolate of *C. albicans* were evaluated [66]. Both compounds triggered a marked decrease in the number of hyphal cells and block hyphal transition along with antibiofilm properties. Another study showed complete *C. albicans* hyphal inhibition with 0.01% camphor with reduced expression of *ECE1* (extent of cell elongation), a hypha-specific gene [123]. 

Biatriosporin D is a phenolic compound isolated from an endolichenic fungus *Biatriospora* sp. It interferes with different aspects of *C. albicans* virulence including adhesion, hyphal and biofilm formation. The authors pointed out to the significant anti-hyphal potential of the compound since it is achieved by much lower dose of Biatriosporin D than its MIC value. The compounds promising antifungal potential was confirmed by in vivo *C. elegans*–*C. albicans* infection model. Exploring its further anti-hyphal mechanisms revealed that Biatriosporin D regulates the Ras1-cAMP-Efg1 pathway in order to inhibit hyphal formation [64]. 

Likewise, another natural product, emodin (1,3,8-trihydroxy-6-methyl anthraquinone), isolated from *Rheum palmatum* rhizomes [74], was shown to reduce fungal hyphal growth in *C. albicans*. Sophorolipids, glycolipid biosurfactants isolated from *Strmerella bombicola* [91], and shikonin, a red pigment isolated from *Lithospermum erythrorhizon* [90], also possessed anti-hyphal potential. Haque et al. [91] explains the inhibitory effect of sophorolipids on hyphal growth with the interfered expression of hypha specific genes *HWP1*, *ALS1*, *ALS3*, *ECE1,* and *SAP4*. 

Eucarobustol E possessed antifungal activity against *C. albicans* and was found to downregulate genes involved in ergosterol biosynthesis. This compound blocks the yeast-to-hypha transition and reduces the cellular surface hydrophobicity of the biofilm cells [75]. Ctn [15–34], the C-terminal fragment of crotalicidin, an antimicrobial peptide from the rattlesnake *Crotalus durissus terrificus* venom, displayed antifungal activity towards *C. albicans*. Both referent and fluconazole resistant *C. albicans*, in planktonic or biofilm form of growth, were susceptible to the peptide application. Range of assays was employed and it was concluded that membrane interaction and disruption underlies peptides antifungal mechanism [54]. 

Other peptide derivatives, cysteine-rich (NCR) peptides NCR335 and NCR169 derivatives, could reduce both biofilm formation and hyphal development of *Candida* strains examined [84]. As recently reviewed, the antimicrobial peptides could have a wide application in both human and veterinary medicine including as antifungals [124].

Morphogenesis in another human fungal pathogen, *C. neoformans*, has different characteristics when compared to the extensively studied other *Candida* species. These fungi change morphology in vivo by inducing a significant increase in the cell size. In vitro, these cells are 4–6 microns big, while their size in vivo is up to 40–50 microns. These cells are called “titan cells”, and, due to their large size, including their capsule, they obstruct the removal by the immune system [125]. According to the study by Folly et al. [95], the antifungal activity of *Xylosma prockia* leaf ethanolic extract and its fractions was investigated for the first time against *C. neoformans* and *C. gattii*. In the mentioned study, morphometric analysis showed that ethyl acetate fraction induces reduced surface/volume ratio for *C. gattii* and *C. neoformans* while also reducing the levels of ergosterol in the fungal cell membrane. Mayer and Kronstad [126] demonstrated inhibitory effect of *Bacillus safensis*, which potently blocked several key *C. neoformans* virulence factors, such as reduction in overall cell size and completely block fungal capsule formation. α-Cyperone, from the rhizome of *Cyperus rotundus*, could be used for the interference with *Cryptococcus* morphology since it reduces the thickness of capsule in *C. neoformans* [97]. 

Eltrombopag, thrombopoietin receptor agonist, was repurposed in order to be used as anti-*Cryptococcus* agent. This drug could be applied as antivirulence agent since it affects cryptococcal virulence factors, such as capsule and biofilm formation, melanin production, and growth ability at 37 °C. Its mechanisms of action include the alteration of genes involved in calcineurin pathway, lipid biosynthesis, membrane component, and transporter genes [73]. Aqueous seed extract from *Allamanda polyantha* could also interfere with *Cryptococcus* morphology by inhibiting capsule formation and decreasing cell size [58].

### 3.3. Mitochondria as Antifungal Targets

Recently, mitochondria have been suggested as potential targets for the development of novel antifungals since they are essential for cell growth and survival of the majority of pathogenic fungi [127].

Treatment of *C. albicans* with rosmarinic acid has significantly reduced the activity of *C. albicans* mitochondria. By employing MTT assay it was suggested that by applying rosmarinic acid (0.1 mg/mL) mitochondrial activity declined for more than 50% [89]. Another natural product, berberine, accumulates in fungal mitochondria and interferes with their activity through impairing mitochondrial membrane potential and mitochondrial Complex I. This compound can also hijack the overexpressed Mdr1p and though reduce antifungal resistance in *C. albicans*. Additionally, it was elucidated that application of berberine was efficient also in vivo, since it can lengthen the survival time of mice with blood-borne dissemination of Mdr1p overexpressed multidrug-resistant candidiasis [63]. 

Papaya, *Carica papaya*, seed extract displayed inhibitory activity towards *C. albicans* by employing different mechanisms, including the accumulation of reactive oxygen species and collapse of mitochondrial membrane potential [68]. Antifungal effect of chiloscyphenol A, a natural small molecule isolated from Chinese liverworts, is highlighted by minimal inhibitory concentration of 8–32 μg/mL and fungicidal activity in both the planktonic state and mature *C. albicans* biofilms. Chiloscyphenol A fungicidal effect was confirmed in vivo on *Caenorhabditis elegans.* This compound induces mitochondrial membrane potential hyperpolarization, increased ATP production and intracellular ROS accumulation, and aggregated distribution of Tom70 indicating a mitochondrial dysfunction as a mechanism of activity. Likewise, it also affects cell membrane integrity as indicated by specific staining and confocal microscopy, transmission electron microscopy (TEM) and by calcein-leakage measurements and flow cytometry [70]. 

Kalopanaxsaponin A, triterpenoid saponin from the stem bark of *Kalopanax pictus*, accomplishes its antifungal effect by increasing the generation of ROS, redundant ROS interferes with respiratory chains and subsequently induce dysfunction of mitochondria. Fungicidal activity of kalopanaxsaponin A is also based on the interference with the permeability of the cell membrane [77].

Mefloquine derivatives in their sub-inhibitory concentrations inhibit the expression of fungal virulence attributes, such as *C. albicans* filamentation and *C. neoformans* capsule formation/melanization. Mechanism of action employed by these derivatives is multitarget and based on the disturbance of both mitochondrial and vacuolar functions [79]. The compound arylamidine T-2307 can selectively disrupt mitochondrial activity in yeast by the mechanism based on inhibition of respiratory chain complexes. This compound acts by inhibiting respiratory chain complexes III and IV both in *Saccharomyces cerevisiae* and in *C. albicans* and subsequently induces a decrease in the intracellular ATP levels in yeast cells. T-2307 is a selective inhibitor and has little effect on bovine respiratory chain complexes [60]. Peptide ToAP2D exhibited antifungal activity towards *Sporothrix globosa;* it inhibited growth of *S. globose*, enhanced apoptosis and induced deformation and rupture of fungal cells. Treatment with ToAP2D reduced the levels of mitochondrial membrane potential and increased ROS levels. Its antifungal potential was confirmed in the in vivo study on mice [93].

### 3.4. Affecting Different Metabolic Pathways and Enzymes in Fungi

Different metabolic pathways could be targeted in order to develop novel antifungal strategies. These pathways include N-acetylglucosamine metabolism, trehalose metabolism, lipid biosynthesis, and other essential processes in fungal cells [128,129]. Likewise, different enzymes in the fungal cells are among the potential targets for drug development, since they are indispensable for fungal growth and virulence [130]. Range of products has been examined as inhibitors of metabolic pathways and/or fungal enzymes with some of them presented in Table 1.

Antimicrobial peptide, AMP-17, interferes with essential *C. albicans* metabolic pathways, including those related to oxidative phosphorylation, RNA degradation, propanoate metabolism, and fatty acid metabolism [53]. Apolipoprotein B (ApoB) derived peptides also displayed antifungal properties. The peptides have slowed down metabolic activity of *Aspergillus niger* spores and interfered with *C. albicans* cell membrane integrity [57]. Among the promising antifungal targets is also enzyme class II fructose-1,6-bisphosphate aldolase (FBA-II) since they are not present in animals and higher plants and though could provide selectivity. 

Novel FBA-II inhibitors were examined towards *C. albicans* with the most promising inhibitory potential observed for the compounds belonging to phenylhydrazones. The assays studying enzyme inhibition highlighted promising activity of these compounds with IC_50_ < 10 μM [87]. Thiazolidinones were analyzed by molecular docking, molecular dynamics, and enzyme inhibition assays and it was shown that they could inhibit enzyme carbonic anhydrase (K_I_ values 0.1–10 µM against the *Candida glabrata* enzyme) [92]. The investigation of Venkata et al. [131] and Ansari et al. [81] evaluated inhibition of glyoxylate cycle by vanillin and monoterpenoid perillyl alcohol against *C. albicans*, respectively.

α-bisabolol suppresses fungal ∆24-sterol methyltransferase. It reduces ergosterol synthesis (26.31–73.77%) dose-dependently and suppresses the expression of *ERG6* by 76.14% at the highest concentration of 9 mM [96]. (S)-2-amino-4-oxo-5-hydroxypentanoic acid is a known homoserine dehydrogenase inhibitor. It was isolated from *Streptomyces* species and found to act as an enzyme-assisted suicide inhibitor of Hom6p. RI-331 possessed activities against *C. albicans*, *C. tropicalis,* and *C. glabrata*, but has no effect against *Aspergillus* species [51].

### 3.5. Fungal Cell Wall

The fungal cell wall has a different structure in relation to human cells and this unique structure is an appealing target for the development of antifungal drugs [132]. The fungal cell wall composition varies between species, but the major constituent is β-(1,3)-glucan polysaccharide covalently cross-linked to chitin, forming the primary scaffold structure [133]. Chitin is synthesized by chitin synthase enzymes Chs1 to Chs3 in *S. cerevisiae*, Chs8 in *C. albicans* and the genes encoding these enzymes are *CHS1*, *CHS2*, *CHS3* and *CHS8,* respectively. 

Chitin synthase enzymes are synthesized in the cytoplasm and then transported to the cell membrane for chitin synthesis [134,135]. Cell wall mannoproteins can be used as potential antifungal agents, though with relatively less success compared to the development of drugs that target β-1,3-glucan synthesis. One of the main components of the cell wall mannoproteins are glycosylphosphatidylinositol (GPI)-modified proteins, including proteins with glycosylhydrolase, glycosyltransferase, or transglycosidase activities. Those enzymes are included in cell wall synthesis, thus are attractive antifungal targets since inhibiting their synthesis and localization to the cell wall could stroke on virulence and cell wall biosynthesis significantly [136].

Some of the antifungal drugs on the market are already based on the inhibition of different aspects of cell wall synthesis. For example, Nikkomycin Z inhibits formation of the chitin structure, process essential for fungi, but not mammals [137]. The recent review has given the overview of natural products targeting the synthesis of β(1,3)-D-glucan and chitin of the fungal cell wall. β(1,3)-D-glucan synthases inhibitors described belong to cyclic lipopeptides, glycolipids and acidic terpenoids [133]. 

Although inhibition of β(1,3)-D-glucan synthases is known mode of action for range of echinocandins that are currently on the market, research of novel inhibitors is continuing up to today. Ibrexafungerp (formerly SCY-078 or MK-3118) is known as a first-in-class antifungal triterpenoid inhibiting the biosynthesis of β-(1,3)-D-glucan in the fungal cell wall. Ibrexafungerp and echinocandin target sites on the β-(1,3)-D-glucan synthase are not the same, implying very limited cross-resistance between echinocandin- and ibrexafungerp-resistant strains [138].

Ge et al. [139] demonstrated that the 3-substituted amino-4-hydroxycoumarin derivatives could reduce chitin level. Antifungal agents that can inhibited in the GPI anchor synthesis are APX001A (pyridine 2-amine-based molecule), phenoxyacetanilide (gepinacin), OGT2468 (rhodamine-3-acetic acid derivatives) and M720. They are productive at low concentrations against *Fusarium*, *Aspergillus*, *Candida*, and *Scedosporium* species. APX001A is effective against caspofungin-resistant *C. albicans* and *C. auris* [136,140]. Magnoflorine (50 μg/mL) inhibited more than 50% of the activity of alpha-glucosidase, an enzyme essential for normal cell wall composition and *C. albicans* virulence, also displaying antibiofilm potential [78].

### 3.6. Antifungal Vaccines

One of the solutions for patients struggling with a high risk of candidiasis might be the prophylactic vaccination—long-term measure used to reduce the frequency of *Candida*-related infections. There are different challenges in this area of research including fungal extensive variation and plasticity, the presence of preestablished immunological tolerance as well as the challenges in advancing protective memory responses in patients strugling with impaired adaptive immunity [141]. 

In vivo studies have been undertaken on mouse, implying efficacy of vaccines protection against fungal pathogens, including species of *Candida*, *Cryptococcus*, and *Aspergillus*. Supporting results have been gathered by using vaccines made of live-attenuated and killed fungi, crude extracts, recombinant subunit formulations, as well as vaccines with nucleic acid as recently reviewed [142].

Immunotherapeutic peptide vaccine-based approach was used in the in silico study that screened *Candida* proteome and identified the most immunodominant HLA class I, HLA class II and B cell epitopes. The authors have selected the most promising epitopes, and created a multivalent recombinant protein against *C. albicans* (mvPC) with added synthetic adjuvant (RS09) in order to increase immunogenicity. The selected mvPC epitopes are homologous against all currently available annotated reference sequences of 22 *C. albicans* strains, suggesting that a higher coverage can be achieved along with greater protective response. mvPCs multivalent nature by recognizing multiple-epitopes could provide enhanced protection against complex *Candida* antigens [83]. 

Other study has employed bioinformatics in order to detect the existence of *C. auris* adhesin proteins, with sequence and structural homologies of Als3, the major *C. albicans* adhesin/invasin. When mice were vaccinated with NDV-3A (a vaccine based on the N-terminus of Als3 protein formulated with alum) they have produced anti-Als3p antibodies that recognized *C. auris* in vitro, disturbed its ability to form biofilms and enhanced macrophage-mediated killing of the fungus. 

Likewise, NDV-3A vaccination induced significant levels of antifungal immune responses, and protected immunosuppressed mice from *C. auris* infection [85]. Vaccination of mice with Sap2-parapsilosis, recombinant secreted aspartyl protease (rSap2) obtained from *Candida parapsilosis*, leads to the increase in survival during systemic *C. tropicalis* infection. This vaccine induces both humoral and cellular immunity, and it provides higher titers of Sap2-induced antibodies that are useful for combating systemic candidiasis [88]. Studies on anti-*Aspergillus fumigatus* vaccine have also been conducted. 

Single immunization with vaccine AF.KEX1 and the squalene:water adjuvant Titermax is able to provide sufficient amount of AF.KEX1 antibodies. By employing techniques, such as qPCR and histological quantification, it was estimated that fungal burden was drastically reduced in the lungs of mice vaccinated with AF.KEX1 and associated levels of protection reported were 75–100% [52]. The *Cryptococcus* vaccine was developed based on the F-box protein Fbp1, a subunit of the SCF(Fbp1) E3 ligase, the protein that increases fungal virulence by regulating host-*Cryptococcus* interactions. 

It is proposed that boosted immunogenicity induced by heat-killed *fbp1*Δ yeast cells could provide protection against a subsequent infection with the virulent parental strain. Heat-killed *fbp1*Δ cells also protected mice depleted of CD4^+^ T cells and provided significant cross-protection against range of invasive fungal pathogens, such as *Cryptococcus neoformans*, *Cryptococcus gattii*, and *A. fumigatus*, along with partial protection against *C. albicans* [76].

## 4. In Vivo and Clinical and Studies of Products Employing Novel Antifungal Pathways

In the modern world, there are available diverse in vivo models for studding fungal infection and subsequently also antifungal agents. One of the in vivo models is *Caenorabditis elegans.* The advantages of using this nematode includes lack of ethics requirements, fully sequenced genome, availability of genetic mutants, easy laboratory maintenance, and the possibility of liquid assays for high-throughput antifungal screening. On the other hand, the disadvantages are inability to grow at 37 °C and absence of an adaptive immune response [143]. It is known that selective pressure from fungal pathogens has impact on the invertebrate innate immune response [144].

Likewise, *Drosophila melanogaster* is also used as the fungal infection model—*Toll*-deficient flies can be utilized to study pathogenesis and drug activity in emerging pathogen *C. auris* [145], while mouse are among the most commonly used fungal infection models of mammals [146]. These in vivo models can be used also for the examination of antifungals targeting biofilms [147]. However, despite different in vivo infection models that can be utilized for the examination of novel antifungals, majority of the studies is based on the in vitro assays. Effort is needed in order to introduce wider use of in vivo models, for the most active antifungals from in vitro studies, in order to more profoundly elucidate their antifungal capacity.

There are novel antifungal therapies in clinical development as reviewed recently [148]. The candidates reviewed employ novel modes of action to defeat resistance; they are safer for use and have less of interactions. The novel candidates target different structures, such as cell wall, glucan synthesis, cell membrane [148]. One of the novel candidates, Fosmanogepix is recently reviewed [149]. This compound is studied for the treatment of invasive fungal infections by *Candida*, *Aspergillus* and rare molds and is the N-phosphonooxymethylene prodrug of manogepix, with mechanism of action being inhibition of GPI-anchored wall transfer protein 1 (Gwt1). 

Since this mode of action is novel, manogepix is efficient in combating resistant strains including echinocandin-resistant *Candida* and azole-resistant *Aspergillus*. Fosmanogepix is proven to be in vivo efficient in range of models, such as mouse and rabbit disseminated infection models [149]. Another agent currently studied is Olorofim. This compound is from orotomide class of antifungals that is studied in clinical trials for the treatment of invasive mold infections. Olorofim target is dihydroorotate dehydrogenase, an essential enzyme in the biosynthesis of pyrimidines. Its efficient against range of fungi, including those resistant to azoles and amphotericin B, but lacks activity against yeasts and the Mucorales [150].

Despite some of the clinical studies currently being conducted, we are still in the demand for the development of novel antifungals. Those would preferably be the ones employing novel mechanisms that can be used to combat resistant fungal strains.

## 5. Conclusions and Future Perspectives

The high incidence of fungal infections worldwide is a significant concern for healthcare practitioners and a challenge for the pharmaceutical industry. The antifungal palette available on the market employs the limited antifungal mechanisms to which fungal pathogens have mainly found ways to adapt. Antifungal resistance is one of the major concerns and the reason for pushing forward the research in the field of antifungal drug discovery. 

Besides increasing ineffectiveness of the current antifungal arsenal, we are also familiar with the range of side effects induced by different antifungal drugs. All of the abovementioned indicates the emerging need for more detailed studies on novel antifungal mechanisms and their utilization for the development of novel drugs. Thus far, research in this field has explored different agents, those of natural or synthetic origin, that employ diverse mechanisms in order to accomplish inhibition of fungal growth and subsequent infection development.

The antifungal mechanisms could be related to the different aspects of fungal growth, metabolism or virulence. Growth reduction in pathogenic fungal strains might be accomplished by interference with their metabolic pathways through impairment of their mitochondrial function or their cell wall. Likewise, one of the antifungal strategies could be associated with lowering the fungal ability to develop resistance to the antimicrobial treatment and could be based on the interference with fungal efflux pumps, ergosterol biosynthesis, or Hsp90 protein. 

Additionally, antifungal strategies could be oriented towards intermingling with the complex architecture of biofilms. In addition to the impact on biofilm biomass and viability of fungal cells, antibiofilm aspects are also related to the thickness of extracellular matrix and its components, such as EPS, as well as the ability of yeast cells to form hyphal structures. Likewise, in addition to yeast hyphae formation, the impact on the morphology of other fungal pathogens could also be used. All of these aspects have been extensively studied and a range of agents has been tested. 

The major concern is that activity of these agents has been explored mainly by in vitro tests. Additional experiments utilizing in vivo infection models, along with subsequent clinical studies, are needed as further steps towards the novel antifungal development. Nevertheless, we are surrounded by a range of products that have promise for combating the emerging threat of fungal infections.

## Figures and Tables

**Table 1:** Overview of some of the agents recently explored as antifungals targeting different structures/processes in fungal cells. (-)—not determined.

Product	Target	Assay	Fungi	Active Concentration	Reference
(*S*)-2-amino-4-oxo-5-hydroxypentanoic acid	Homoserine dehydrogenase	Spectroscopic studies, and X-ray crystallography	*Candida albicans*, *C. tropicalis*, *C. glabrata*	64–128 µg/mL	[51]
AF.KEX1	Endoprotease	Mice in vivo study	*Aspergillus fumigatus KexB*	50 µg	[52]
AMP-17	Range of metabolic pathway	Proteomic analysis	*C. albicans* ATCC 10231	32 μg/mL	[53]
Antimicrobial peptide Ctn [15–34]	Biofilm, cell membrane	XTT and fluorescent dyes, CLSM, and atomic force microscopy; Fluorescence assays	*C. albicans* (ATCC 90028) and *C. albicans* LABMIC 0125	10 µM	[54]
Antimicrobial peptide VLL-28	Damaging the cell wall/biofilm	XTT reduction assay, CV assay, CLSM	Range of Candida strains	12.5 –25 µM	[55]
Apigenin, Apigetrin, Luteolin, Quercetin, Quercitrin, Isoquercitrin, Rutin	Hyphal growth, biofilm, CDR1	Microscopic examination, CV antibiofilm assay, qRT-PCR	*C. albicans* (475/15)	0.0375–0.075 mg/mL	[56]
ApoB-derived peptides	Cell membrane, metabolic activity	Propidium iodide intake; cell proliferator reagent WST-1	*C. albicans* ATCC 10231, *A. niger* N402	5–40 µM	[57]
Aqueous seed extract from *Allamanda polyantha*	Capsule formation, size	Fluorescent microscopy	*C. neoformans* H99	281–1126 µg/mL	[58]
*Artemisia absinthium* extract	Biofilm, EPS, cell membrane	CV antibiofilm assay, Congo red assay, nucleotide leakage and CV uptake assays	*C. albicans* (475/15)	0.5 mg/mL	[59]
Arylamidine T-2307	Inhibition of respiratory chain complexes	Assessment of respiratory chain enzymatic activities, measurement of intracellular ATP levels	*Saccharomyces cerevisiae* 24657 (D273-10B) and *C. albicans* MYA-2876 (SC5314)	220–344 µM	[60]
Astragalin	Hyphal growth, cell membrane integrity	Microscopic examination, nucleotide leakage assay	*C. albicans* (475/15)	0.075 mg/mL	[61]
Benzoxazole derivatives	Ergosterol, cell efflux	Ergosterol estimation assay using spectrophotometry and HPLC, Rhodamine 123 efflux	*C. albicans* (SC5314)	0.125–160 µg/mL	[62]
Berberine	Mitochondrial function and Mdr1p	RNA-seq	*C. albicans* CaS and CaR	32 μg/mL	[63]
Biatriosporin D	Hyphal growth	Microscopy, qRT-PCR	*C. albicans* (SC5314 and BWP17-DPP3-GFP)	0.5–4.0 μg/ml	[64]
Blue light	Biofilm viability	Adherence inhibition, developmental inhibition, and disruption biofilm assays (CFU count LIVE/DEAD BacLight viability staining)	*C. albicans* (SC5314) and *C. auris* (AR0383, AR0389, and AR0390)	-	[65]
Camphor Eucalyptol	Hyphal growth and biofilm	Microscopy, CV antibiofilm assay	*C. albicans* (475/15)	0.125 mg/mL and 23 mg/mL	[66]
Cannabidiol	EPS production	Confocal laser scanning microscopy (CLSM), qRT-PCR	*C. albicans* SC5314	50 µg/mL	[67]
*Carica papaya* seeds extract	Mitochondrial function	Measurement of mitochondrial membrane potential, assay for mitochondrial enzyme activities	*C. albicans* ATCC	5–25 µg/mL	[68]
Catechol	Hyphal growth, biofilm, downregulation of RAS1, HWP1 and ALS3	Microscopy, CV assay, RT-PCR	*C. albicans* (ATCC 10231)	2–256 μg/mL	[69]
Chiloscyphenol A	Mitochondrial function	Analysis of mitochondrial membrane potential, measurement of intracellular ATP production, observation of the localization of Tom70-GFP	*C. albicans* (SC5314)	Up to 64 μg/mL	[70]
Curcumin-sophorolipid nanocomplex	Biofilm and hyphae	CV assay, CLSM, qRT-PCR	*C. albicans* (SC5314)	9.37 μg/mL	[71]
Dioscin	Hyphal growth, biofilm, production of phospholipase	Microscopy, XTT reduction assay, CLSM, Egg yolk phospholipase assay	*C. albicans* (SC5314)	4 μg/mL	[72]
Eltrombopag	Capsule and biofilm formation, melanin production	ImageJ of stained cells, XTT reduction assay, L-DOPA melanization assay	*C. neoformans* H99	0.06 mg/l	[73]
Emodin	Hyphal growth and biofilm formation	Morphology on Spider medium, MTT assay	*C. albicans* (ATCC 10231)	12.50 μg/mL	[74]
Eucarobustol E	Hyphae, biofilm	Filmentation assay on hypaae inducing medium, XTT reduction assay, CLSM, SEM, RNA-seq, RT-PCR	*C. albicans* (SC5314 and ATCC 24433)	8.0–16.0 μg/mL	[75]
Heat-killed fbp1Δ cells	F-box protein Fbp1, ligase subunit	Mice in vivo study	*Cryptococcus neoformans* H99 and its mutants, *C. gattii* R265 and *C. albicans* SC5314, *A. fumigatus* R21	5 × 10^7^ heat-killed fungal cells	[76]
Kalopanaxsaponin A	Mitochondrial function and cell membrane	Asessment of alteration of mtΔψ and ATP content, PI staining, TEM	*C. albicans* (SC5314)	8–32 µg/mL	[77]
Magnoflorine	Alpha-glucosidase	Spectrophotometric alpha-glucosidase inhibition assay	*C. albicans* (ATCC 10231)	50 µg/mL	[78]
Mefloquine derivatives	Mitochondrial and vacuolar function	MitoTracker Red uptake	*C. albicans* (SC5314), *C. neoformans* H99	1–64 μg/mL	[79]
Mohangamide A	Isocitrate lyase in glyoxylate pathway	High-Throughput Screening	*C. albicans* (SC5314)	4.4 µM	[80]
Monoterpenoid perillyl alcohol	Glyoxylate cycle inhibitor	Isocitrate lyase enzyme assay	*C. albicans* (SC5314)	320 µg/mL	[81]
Morin	Hyphal growth, biofilm formation, phospholipase and exopolysaccharide production	Microscopic analysis; CV antibiofilm assay; Egg yolk assay; Fourier Transform Infrared Spectroscopy Analysis	*C. albicans* (ATCC 90028)	150 μg/ml	[82]
mvPC	Multivalent	In silico study	*C. albicans* (SC5314)	-	[83]
NCR Peptide Fragments	Biofilm, hyphae	XTT reduction assay; Microscopy	*C. albicans* ATCC 10231, *C. tropicalis* CBS 94	0.78–12.5 µM	[84]
NDV-3A	Adhesins	In silico and in vivo mice studies	*C. auris* strains (CAU-01, CAU-03, CAU-05, CAU-07, and CAU-09)	300 μg	[85]
*Ononis spinosa* methanolic extract	Biofilm, cell membrane	CV antibiofilm assay, nucleotide leakage assay	C. albicans (ATCC 10231)	10 mg/mL	[86]
Phenylhydrazones	Fructose-1,6-bisphosphate aldolase	Molecular dynamics, enzyme inhibition assay	*C. albicans* (SC5314)	2.7–4.1 µM	[87]
Recombinant Sap2	Secreted aspartyl protease	Mice in vivo study	*C. tropicalis* (ATCC 750)	10 μg	[88]
Rosmarinic acid	Biofilm, EPS production, mitochondrial activity	CV antibiofilm assay, Congo red binding assay, MTT assay	*C. albicans* (475/15)	0.025–0.1 mg/mL	[89]
Shikonin	Hyphal growth, biofilm formation	Microscopy, XTT reduction assay, CLSM	*C. albicans* (SC5314)	0.5–4 μg/mL	[90]
Sophorolipids	Biofilm and hyphae	XTT reduction assay, SEM and CLSM analysis, qRT-PCR	*C. albicans* (SC5314)	15.0–30.0 μg/ml	[91]
Thiazolidinones	Carbonic anhydrase	Molecular docking and molecular dynamics, enzyme inhibition assay	*Candida glabrata*	0.1–10 µM	[92]
ToAP2D Peptide	Mitochondria	JC-1 kit	*Sporothrix globosa*	4 mg/mL	[93]
Usnic acid	EPS production	Spectrophotometrically, FT-IR analysis	*C. albicans* (ATCC 90028)	100 μg/ml	[94]
*Xylosma prockia* ethyl acetate fraction	Ergosterol	Spectrophotometric quantification	*C. gattii* (ATCC 24065 and ATCC 32608) *C. neoformans* (ATCC 24067, ATCC 28957, ATCC 62066, and ATCC H99)	1–16 mg/L	[95]
α-bisabolol	Ergosterol production	Spectrophotometry method	*A. fumigatus* (Af 239)	0.28–9.0 mM	[96]
α-Cyperone	Capsule	Microscopic examination	*C. neoformans*	16 µg/mL	[97]

## Data Availability

The data presented in this study are available in this article.
